# Using mesh in capsule anatomical reconstruction to enhance the stability of high-dislocation-risk hip arthroplasty: a randomized controlled trial

**DOI:** 10.1186/s13018-023-03575-1

**Published:** 2023-02-14

**Authors:** Peng Xin, Ming Ni, Quanbo Ji, Xiaoxi Yang, Lei Geng, Yan Wang, Guoqiang Zhang

**Affiliations:** 1 Medical School of Chinese People’s Liberation Army, Beijing, 100853 China; 2 Department of Orthopedics, The First Medical Center of Chinese PLA General Hospital, Fuxing Road, Haidian District, Beijing, 100048 China; 3 Senior Department of Orthopedics, The Fourth Medical Center of Chinese PLA General Hospital, Beijing, 100048 China; 4 Department of Orthopedics, Chinese PLA Southern Theater Command General Hospital, Guangzhou, 510010 China; 5 Department of Orthopedics, Peking University Third Hospital, Beijing, 100191 China

**Keywords:** Total hip arthroplasty, Capsular repair, Mesh, Hip dislocation

## Abstract

**Background:**

Dislocation is a common complication after total hip arthroplasty (THA). This study aimed to compare the outcomes of mesh reconstruction versus conventional capsular repair in maintaining capsular integrity and preventing dislocation after THA.

**Methods:**

This was a prospective, randomized controlled study of consecutive patients. A total of 124 high-dislocation-risk THAs were identified and randomized into two groups, one using mesh reconstruction and the other using the conventional capsular repair method. Perioperative data and radiological data were collected. Patients were followed up regularly. The main indices were the capsular integrity assessed by magnetic resonance imaging (MRI) and hip dislocation rate. The secondary indices included the Harris hip score (HHS), complications, and satisfaction.

**Results:**

A total of 106 patients completed the follow-up and the average follow-up times were 19 ± 3.1 and 18 ± 3.3 months. The operation time of the mesh group was longer than that of the conventional group (*P* < 0.001). There were minor differences in acetabular anteversion and abduction angle, and the other data showed no differences. MRI results indicated that the success rate of capsular repair was higher in the mesh group (50 hips, 98%) than in the conventional group (37 hips, 67%) (*P* < 0.001), and the others failed the repair. Three dislocations occurred in the conventional group, while none occurred in the mesh group. The preoperative HHS (30 points) and postoperative HHS (82 points) of the mesh group were similar to those (35 points, 83 points) of the conventional group (*P* = 0.164, *P* = 0.328). Satisfaction had no difference (*P* = 0.532).

**Conclusions:**

Compared to conventional repair, mesh reconstruction can effectively maintain capsular integrity and decrease dislocation risk after THA without increasing complications.

*Level of evidence*: Therapeutic study, Level IA.

## Introduction

Dislocation is one of the most common complications after primary total hip arthroplasty (THA), with an incidence of 0.2–10% [1, 2]. More than 60% of these patients with dislocations suffered multiple occurrences, and more than half required revision surgery [3]. Several capsular repair techniques have been used to decrease the risk of complications, but the optimal option remains controversial [2, 3].

The presence of capsular dehiscence appears to have an effect on the incidence of postoperative posterior implant dislocation [4–6]. Capsular repair is a common surgical technique used to decrease the risk of dislocation after primary hip arthroplasty [7]. Mihalko and Whiteside [8] found that external rotators and capsular repair produced a nearly normal load‒deflection curve. Chou et al. [9] conducted a meta-analysis and found that capsule repair was more effective in reducing the risk of dislocation than rotator repair. In a cadaver study, Takao et al. [10] found that the capsule significantly contributes more than the external rotator to hip stability. Many researchers believe that the capsule plays an important role in the biomechanical stability of the hip [2, 11–14]. However, repair failure may occur due to limited capsular tissue, and approximately half of hips fail to achieve adequate stability due to capsular dehiscence [6, 15, 16]. Larger hip offset, longer repair distance and earlier exercise increased the incidence of repair failure. These drawbacks can be avoided by using artificial synthetic mesh [17]. Masterson et al. [18] used mesh to prevent hip dislocation after extensive resection of proximal femoral tumours and prosthesis reconstruction, but reconstruction efficiency has not been fully evaluated. Currently, the efficiency can be assessed using magnetic resonance imaging (MRI) [15, 19].

This randomized prospective study aimed to compare mesh reconstruction with conventional capsular repair in maintaining capsular integrity and preventing hip dislocation after primary THA, and to evaluate the safety of mesh reconstruction. The hypothesis was that mesh reconstruction was superior to conventional capsular repair.

## Materials and methods

This trial was approved by the Institutional Review Board and the Hospital Ethics Committee. The study was registered at the Chinese Clinical Trial Registry. Informed consent was obtained from each patient.

From September 2020 to October 2021, patients undergoing hip arthroplasty in our hospital were included in this study. The eligibility of the study included: (1) adult patients; (2) primary hip arthroplasties; and (3) risk factors for hip dislocation, including neuromuscular disease (poliomyelitis, stroke sequelae), multisegmental lumbar spine fusion, obesity (body mass index ≥ 35 kg/m^2^), and poor lower extremity muscle strength (muscle strength of any one of the iliopsoas, quadriceps, gluteus medius and gluteus maximus muscles was less than grade 4 by testing hip flexion, abduction, and extension) [20–24]; (4) patients who were willing to participate in the study. The exclusion criteria were: (1) preoperative surgical site infections; (2) confirmed allergies to mesh; (3) skeletal abnormalities of lower limbs: type 3 or above developmental dysplasia of the hips, rheumatoid arthritis, ankylosing spondylitis, and so on; (4) the joint capsule was damaged or resected in the past; and (5) patients with poor compliance or serious diseases, such as severe diabetes, taking influential drugs and so on.

The sample size was calculated based on the preliminary results of previous studies. The success rate of the mesh group (treated with mesh reconstruction) was 90% in the preliminary experiment, and the success rate of the conventional group (treated with the tendon-to-bone capsular repair technique) was 65% [15]. We selected confidence (1 − *β*) = 0.80, significance level *α* = 0.025 (unilateral), and superiority margin *δ* = 0.025. Using PASS 13.0 software, we calculated the sample size of 50 patients and expanded the sample size to 62 patients in each group, considering a 20% dropout rate. There were 2 groups, requiring 124 patients.

**Fig. 1:**
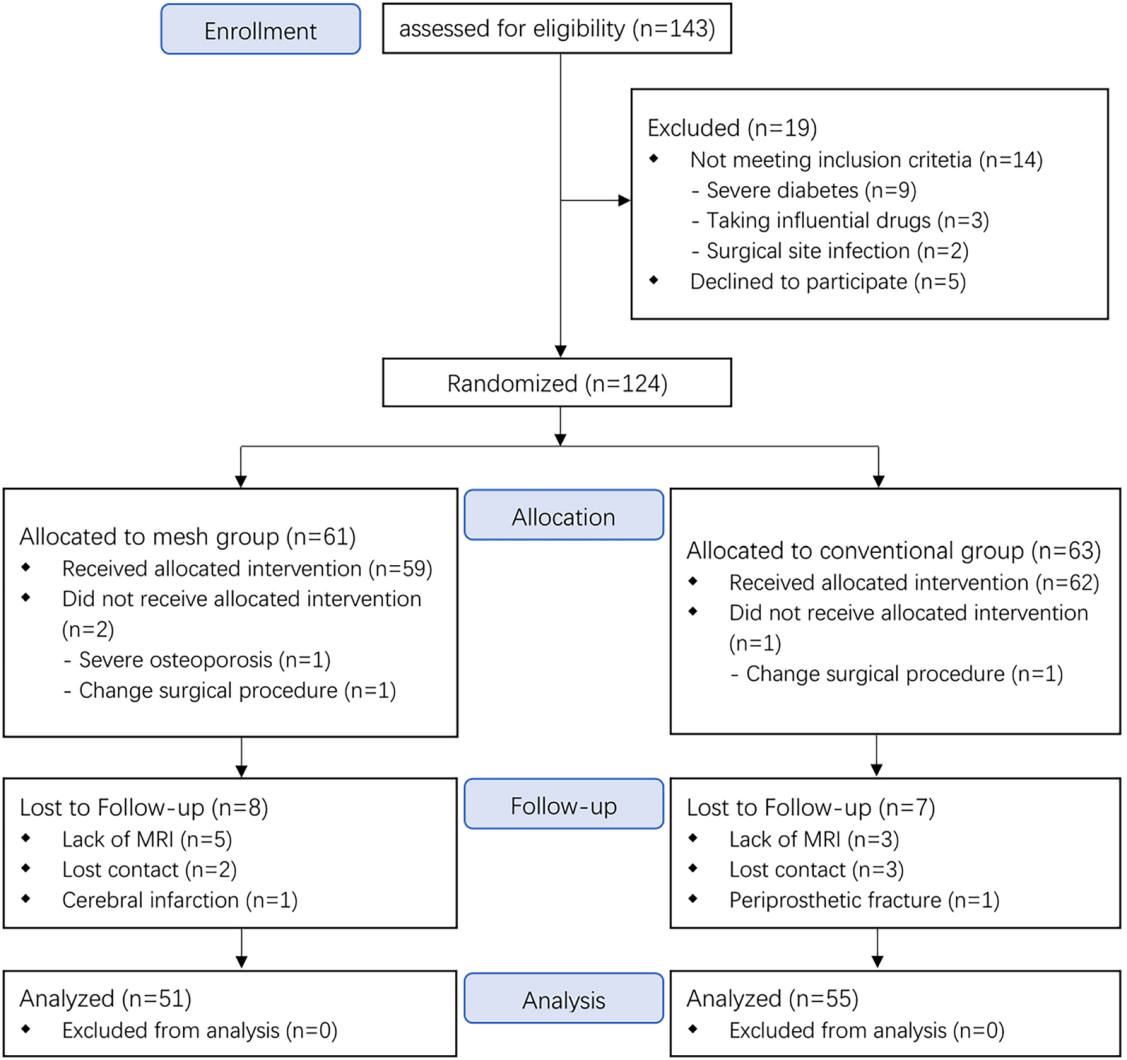
CONSORT (Consolidated Standards of Reporting Trials) flowchart of enrollment and analysis

A total of 124 patients were enrolled and randomly assigned into two groups. An assistant who did not participate in the study performed the randomization using a computer-generated random-number table. These numbers were sealed in sequentially numbered opaque envelopes opened by a nurse in the operating room. The surgeons performed either mesh reconstruction or conventional capsular repair methods based on the numbers. All primary THAs were performed by two senior surgeons using the posterolateral approach and the same surgical technique. For all patients, preoperative templating was performed to prepare the appropriate implants. The implants were Pinnacle cups and Tri-lock stem, Corail stem, Corail Revision stem (for patients with severe osteoporosis or huge medullary cavity) (Depuy, America). Patient enrolment is shown in Fig. 1. Nineteen patients were removed from the study cohort due to unreceived allocated intervention or incomplete follow-up. There were 51 patients (51 hips) in the mesh group and 55 patients (55 hips) in the capsular group (Table 1).

**Table 1:** Pre-operation demographics data

Variables	Mesh reconstruction group (*n* = 51 Hips)	Conventional capsular repair group (*n* = 55 Hips)		*P* value
	*n* (%) or Mean ± Standard deviation			
Age at surgery (y)*	73.9 ± 4.78	74.5 ± 6.19	*t* = − 0.527	0.599
BMI (kg/m^2^)*	25.1 ± 3.16	24.9 ± 3.37	*t* = 0.191	0.849
Sex^†^			*χ*^2^ = 0.907	0.341
Male/female	15 (29%)/36 (71%)	21 (38.2%)/34 (61.8%)		
Side†			*χ*^2^ = 0.593	0.441
Left/right	24 (47%) / 27 (53%)	30 (54%) / 25 (46%)		
ASA score^†^			*χ*^2^ = 1.298	0.255
2 /3	37 (73%) / 14 (27%)	45 (82%) / 10 (18%)		
Reason for hip arthroplasty^†^				
Femoral fracture	14 (28%)	11 (20%)	*χ*^2^ = 0.055	0.815
Osteonecrosis	21 (41%)	26 (47%)	*χ*^2^ = 0.398	0.528
Osteoarthritis	16 (31%)	18 (33%)	*χ*^2^ = 0.022	0.881
Risk for dislocation^†^				
Neuromuscular disease	9 (18%)	13 (24%)	*χ*^2^ = 0.577	0.447
Multi-segmental lumbar spine fusion	22 (43%)	19 (34%)	*χ*^2^ = 0.824	0.364
Obesity (BMI ≥ 35 kg/m^2^)	17 (33%)	18 (33%)	*χ*^2^ = 0.004	0.947
Poor muscle strength (< grade 4)	3 (6%)	5 (9%)	*χ*^2^ = 0.390	0.532

BMI, Body mass index, ASA, American society of anesthesiologists. THA, total hip arthroplasty

*The values are given as the mean and standard deviation

^†^The values are given as the number with the percentage in parentheses

### MESH group

The operation was performed under general anaesthesia. The patient was placed in the lateral decubitus position. The operation was performed through the posterolateral approach (appoximately 15 cm in length). The piriformis tendon and conjoined tendon (both obturator and both gemellus muscles) were detached near their insertions. The tendons were pulled posteriorly to expose the posterior capsule. Then, the posterior capsule was detached from the femoral neck and turned over to the acetabular labrum. The femoral head was dislocated posteriorly from the acetabulum. A proper uncemented acetabular cup and femoral stem were implanted. Based on the preoperative digital templating, the acetabular cup was placed at 20° ± 5° of anteversion and 40° ± 5° of abduction, and the stem was placed at 15° of anteversion. Hip stability was tested with the hip and knee flexed at 90° and the leg internally rotated by 25°. The combined anteversion of the implants was assessed with the Ranawat sign [25, 26].

**Fig. 2:**
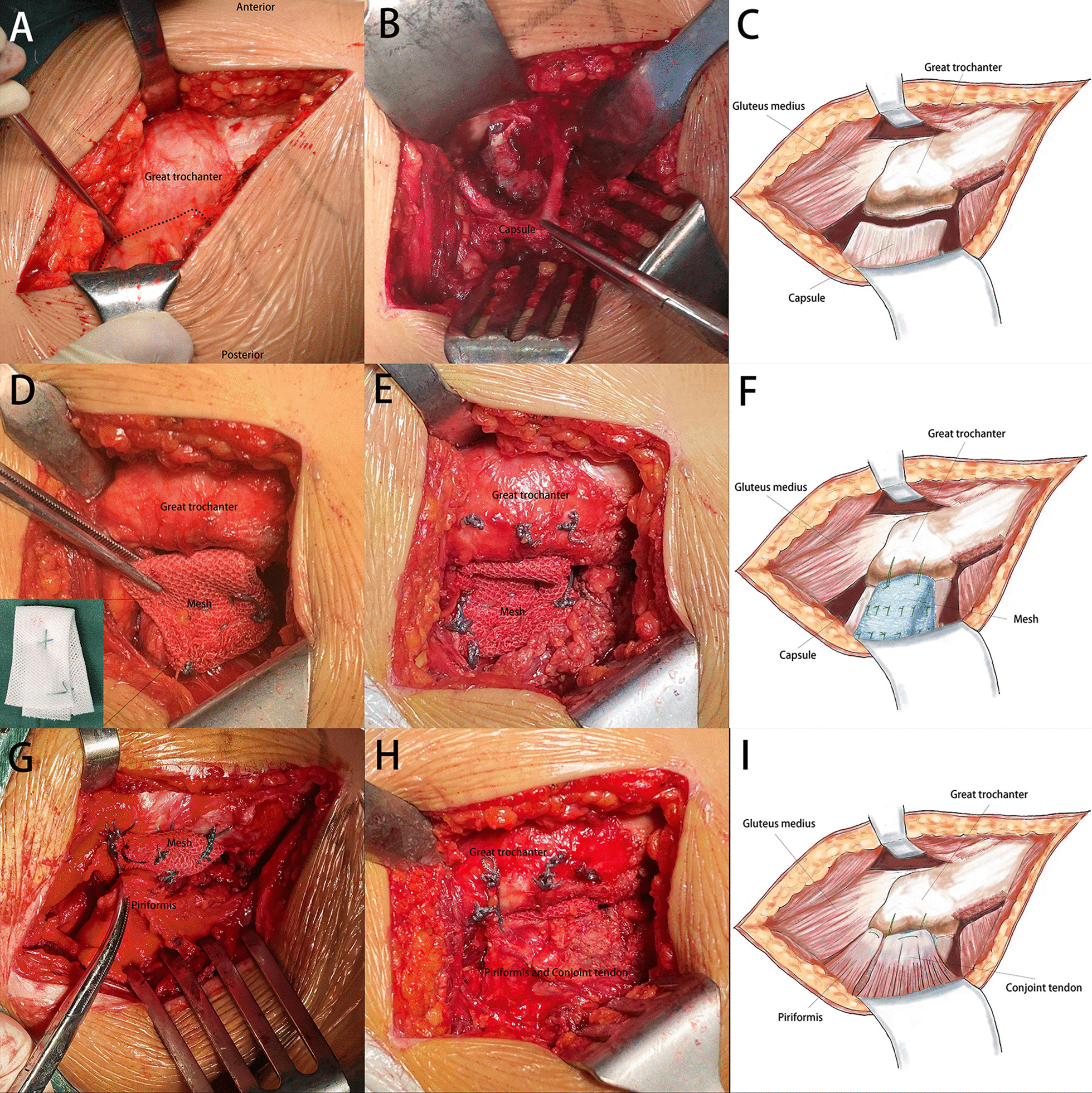
The posterior soft tissue was separated along the black dashed line (**A**). The posterior capsule was detached from the femoral neck (**B**), as illustrated (**C**). The mesh was sutured to the capsule (**D**) and then sutured to the greater trochanter (**E**), as illustrated (**F**). The piriformis and conjoint tendon were transferred over the mesh (**G**) and sutured to the greater trochanter (**H**), as illustrated (**I**)

The hydrophilic three-dimensional mesh (15 cm × 10 cm; TET1510, Parietex, COVIDIEN, USA) was folded into a V-shaped pattern. We made two or three drillholes (1 cm apart) in the trochanteric crest of the great trochanter. With the hip flexed at 90°, the two ends of the mesh were tensioned and sutured to the posterior capsular attachment using nonabsorbable sutures (MB66, Ethibond, USA) passed through the drillholes. The apex of the V-shaped mesh was sutured to the greater trochanter using MB66 nonabsorbable suture. The spare mesh was removed with scissors. After acceptable hip motion and stability were confirmed, the external pronator and piriformis muscles were transferred over the mesh and sutured to the greater trochanter using MB66 sutures (Fig. 2). The wound was closed in the usual manner.

### Conventional capsular repair group

**Fig. 3:**
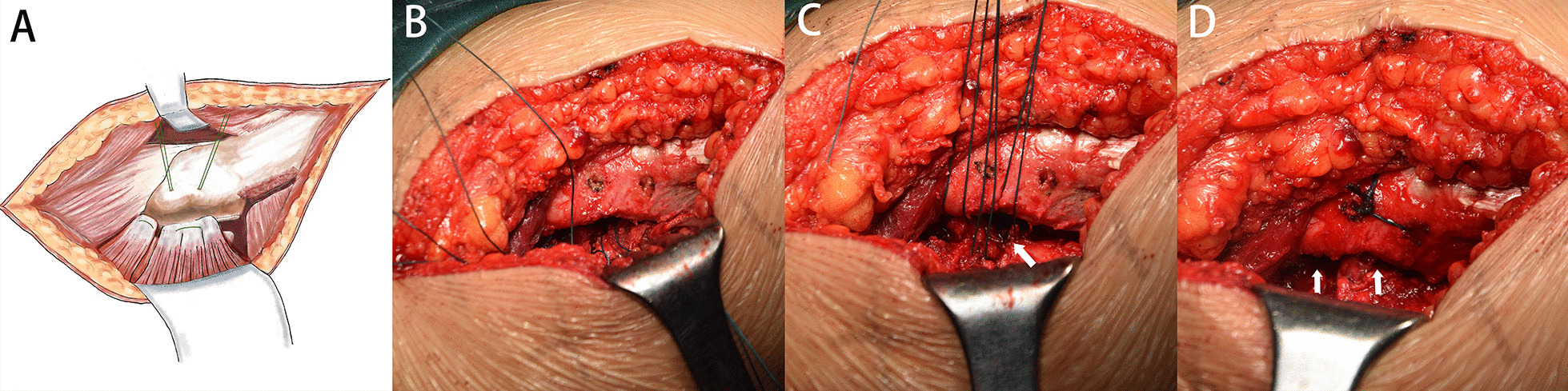
The posterior capsule and external rotator muscle were repaired by conventional tendon-to-bone method as illustrated (**A**). Two drillholes (2 cm apart) were made, and the capsule and tendon were sutured using the modified Kessler method (**B**). When the leg is in the neutral position, the capsule cannot contact the intertrochanteric ridge (white arrows in **C**). After hip movement, the capsule and the femur were not in contact (white arrows in **D**)

The surgical approach and arthroplasty were the same as those in the mesh group. We made two drillholes (2 cm apart) in the trochanteric crest of the greater trochanter. We passed MB66 nonabsorbable sutures through the drillholes and sutured the capsule and short external rotator tendons together with the modified Kessler method [6, 27] (Fig. 3). The wound was closed in the same manner.

### Postoperative management

Full weight-bearing was started on the first postoperative day. Two weeks after surgery, hip range of motion exercises were started, but the hip flexion was less than 90°. Hip adduction and internal rotation were avoided.

### Evaluation

**Fig. 4:**
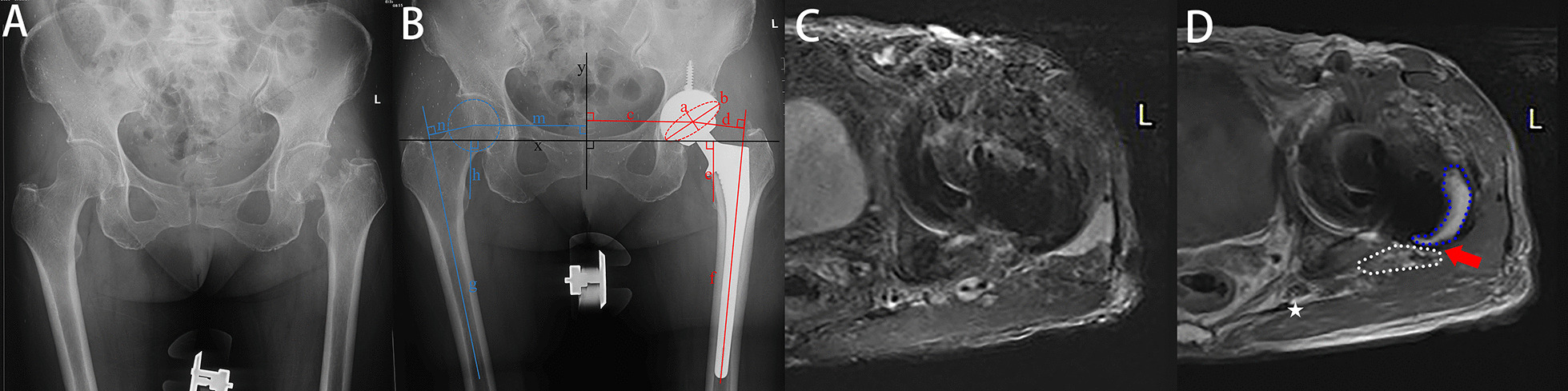
Female, 70 years old, femoral neck fracture (**A**). THA with mesh reconstruction was performed in this patient (**B**). Follow-up MRI (**C**, **D**) at six months after surgery showed that mesh was clung to the posterior femur without loosening sign (red arrows). The blue dashed line is the femur, the white dashed line is the mesh, the pentagram is the sciatic nerve. Detailed radiological measurements are shown in **B** acetabular anteversion angle = sin^−1^ (*a*/*b*); acetabular abduction angle = the angle between *b* and *x*; lower extremity length difference = *e* − *h*; offset difference = *c* + *d* − (*m* + *n*)

An experienced surgeon who did not attend the treatments assessed the outcomes. Perioperatively, all patients underwent preoperative and postoperative radiological evaluations, including a standardized anteroposterior view of the pelvis as well as a lateral view of the affected hip and femur. The anteversion angle is the sine of the minor and long axes in the area of the elliptical projection of the cup. The acetabular abduction angle was defined as the angle between the acetabular long axis and the interteardrop line. The femoral offset is the sum of the distance from the rotation centre to the pelvis midline and the femur. The leg length discrepancy was measured as the difference in the perpendicular vertical displacement from the teardrop line to the lesser trochanter. Detailed measurements are shown in Fig. 4B. All measurements were performed using OrthoView imaging software (Materialize, Ann Arbor, MI). All measurements were scaled (reference: the diameter of the femoral head in X-ray radiography compared to the actual diameter) to determine the true value. The operation time and the level of C-reactive protein (CRP) and erythrocyte sedimentation rate (ESR) can be found in the hospitalization records. These patients returned to the hospital six months after surgery for MRI. All MRIs were performed using the section encoding for metal artefact correction technique (SEMAC, Siemens Healthcare, Erlangen, Germany) [15, 28]. MR images could clearly distinguish the capsule, muscle and bone. MRIs should extend from the acetabulum to the proximal femoral diaphysis, to include the entire capsule. The capsule arises from the edge of the acetabulum and ends at the intertrochanteric line. T1WI and T2WI axial MRIs were used to evaluate the integrity of the capsule. The capsule appeared as low signal intensity on both T1WI and T2WI images. The posterior capsule was considered intact when there was contact of the posterior capsule with the greater trochanter. Failed repair of the posterior capsule is indicated by a gap with fluid signal intensity between the capsule and the greater trochanter [15, 16, 28]. Scar tissue remodelling of the posterior soft-tissue envelope may appear as intermediate to high signal intensity. We assessed the integrity of the mesh repair by measuring the distance between the mesh (low signal intensity on T1WI and T2WI) and the greater trochanter in millimetres. The distance was interpreted as hyperintense scar tissue or fluid filled between the mesh and the greater trochanter. A distance greater than 5 mm was considered a failure of the repair [16]. Hip function was. annually assessed with the Harris hip score (HHS) [29]. Satisfaction was assessed on a 5-point Likert scale.

### Statistical analysis

Continuous variables are presented as the mean and standard deviation (SD), and categorical variables are presented as frequencies and percentages. Categorical variables were analysed with the chi-square test and Fisher’s exact test when proportions were < 5. For data with a normal distribution, continuous variables were analyzed with Student’s t test for data with asymmetrical distribution, and nonparametric Mann‒Whitney tests and sign tests were used in asymmetric distributions. Statistical significance was set at *P* < 0.05. Statistical analyses were performed using SPSS version 22 (Inc., Chicago, IL, USA).

## Results

A total of 106 patients received the allocated intervention and completed more than one-year of follow-up. There were no significant differences between the two groups in the demographic data and baseline assessments (*P* < 0.05) (Table 1).

The mean operative times were 91 ± 14.2 and 78 ± 15.9 min in the mesh and conventional groups, respectively (*P* = 0.006). There were no significant differences in perioperative CRP and ESR (*P* > 0.05). This result implied that mesh did not increase the risk of infection. The mean angles of the Ranawat sign were 33.9 ± 3.53 and 34.5 ± 3.16, respectively (*P* = 0.380). The mean acetabular anteversion angles of the two groups were 19.1 ± 2.76 and 20.4 ± 2.58, respectively (*P* = 0.015). The mean abduction angles were 40.6 ± 2.75 and 38.9 ± 2.59, respectively (*P* = 0.001). These results meant that there were no differences in the position of implants. There were no significant differences in the leg length discrepancy (0.9 ± 1.46 and 0.6 ± 1.54; *P* = 0.300) and the hip offset discrepancy (1.6 ± 2.28 and 2.1 ± 2.46; *P* = 0.229) (Table 2). These results indicate that no differences were seen in the tension of hip soft tissue.

**Table 2:** Perioperative data and radiological results

Variables	Mesh reconstruction group (*n* = 51 Hips)	Conventional capsular repair group (*n* = 55 Hips)		*P* value
	Mean ± Standard deviation			
Operation time (min)	91 ± 14.2	78 ± 15.9	*t* = 4.327	< 0.001
CRP and ESR				
Pre-operation CRP (mg/dl)	0.75 ± 1.17	0.85 ± 1.28	*t* = − 0.443	0.658
Post-operation CRP (mg/dl)	2.96 ± 2.59	3.64 ± 2.68	*t* = − 1.313	0.192
Pre-operation ESR (mm/H)	15.9 ± 11.66	17.5 ± 11.96	*t* = − 0.699	0.486
Post-operation ESR (mm/H)	25.9 ± 15.02	29.3 ± 14.75	*t* = − 1.166	0.246
Ranawat sign (combined ante version) (°)	33.9 ± 3.53	34.5 ± 3.16	*t* = − 0.881	0.380
Radiological evaluation				
Acetabular ante version angle (°)	19.1 ± 2.76	20.4 ± 2.58	*t* = − 2.479	0.015
Acetabular abduction angle (°)	40.6 ± 2.75	38.9 ± 2.59	*t* = 3.308	0.001
Leg length discrepancy (mm)	0.9 ± 1.46	0.6 ± 1.54	*t* = 1.042	0.300
Offset difference (mm)	1.6 ± 2.28	2.1 ± 2.46	*t* = − 1.210	0.229

CRP, C-reactive protein; ESR, erythrocyte sedimentation rate

**Fig. 5:**
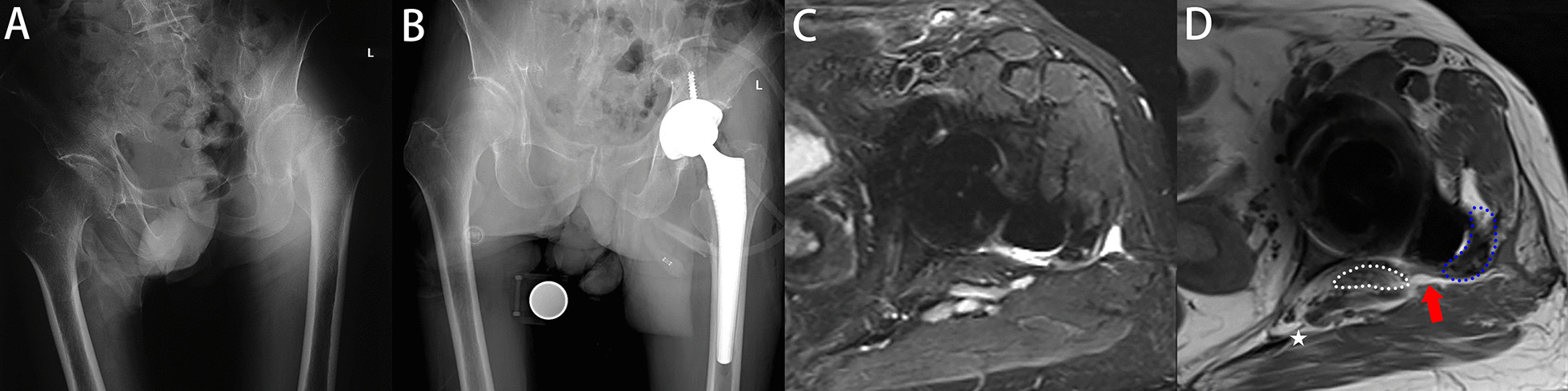
Female, 75 years old, femoral neck fracture (**A**). THA with mesh reconstruction was performed (**B**). Follow-up MRI (**C**, **D**) showed a gap between mesh and femur (red arrows). Symbols are identified as above

**Fig. 6:**
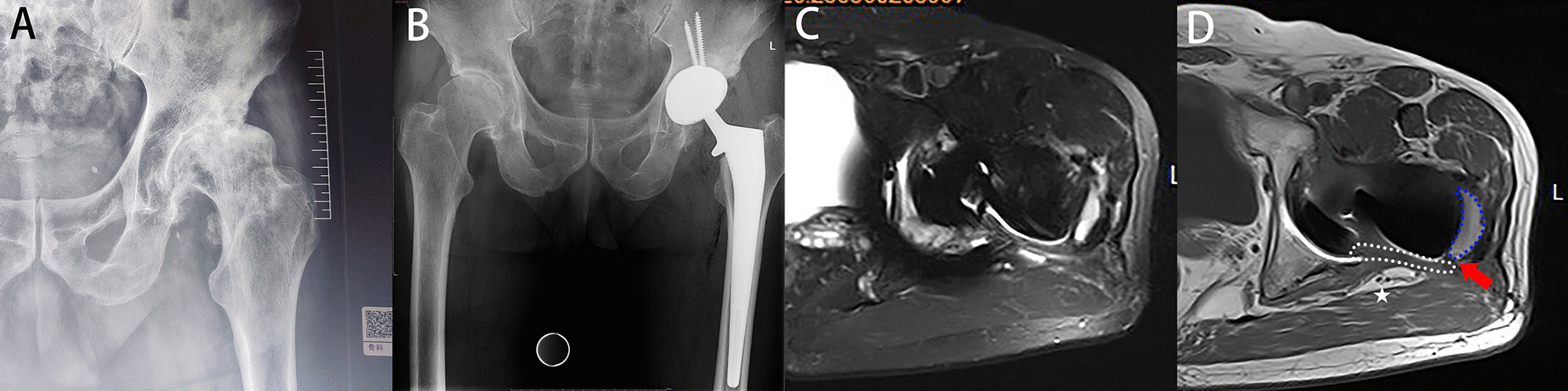
Male, 73 years old, hip osteoarthritis (**A**). THA with conventional capsular repair was performed (**B**). Follow-up MRI (**C**, **D**) showed the capsule was contacted with the greater trochanter. Symbols are identified as above

**Fig. 7:**
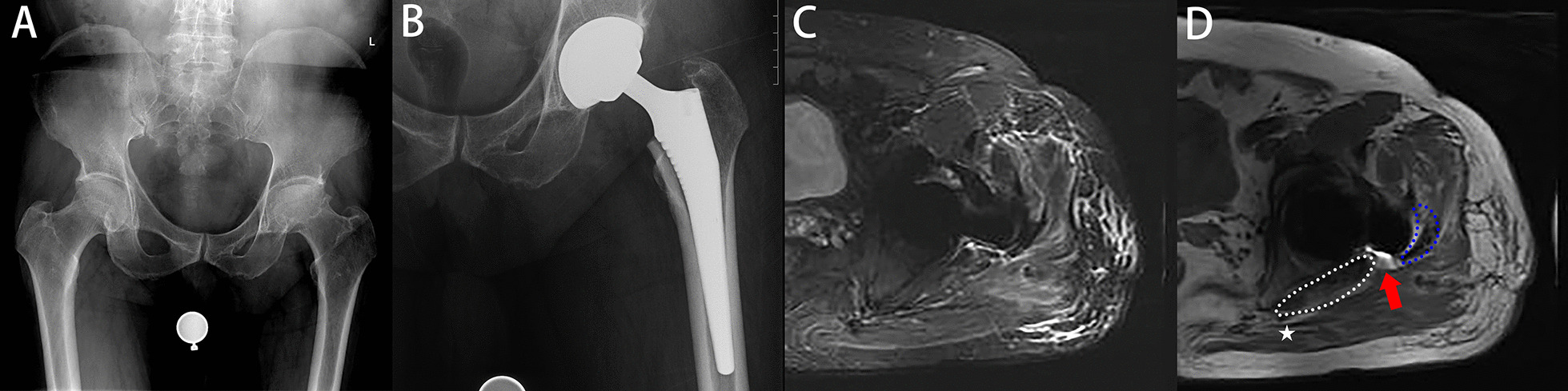
Male, 63 years old, osteonecrosis of femoral head (**A**). THA with conventional capsular repair was performed (**B**). Follow-up MRI (**C**, **D**) showed a gap between capsule and femur (red arrows). Symbols are identified as above

Postoperatively, follow-up lasted for 19 ± 3.1 months in the mesh group and 18 ± 3.3 months in the conventional group (*P* = 0.162). According to the postoperative MRI results, the success rates of capsule repair in the two groups were 98% (50 hips) and 67% (37 hips), respectively (*P* < 0.001) (Figs. 4, 5, 6 and 7). No dislocation occurred in the mesh group (0 hips, 0%), while three patients in the conventional group developed hip dislocation (3 hips, 5%) (*P* = 0.244). At the final follow-up, the postoperative HHS of both groups was higher than the preoperative HHS. There was no significant difference in postoperative HHS between the two groups (82 ± 4.8 and 83 ± 3.9; *P* = 0.328). There were no complications except dislocation in either group, such as sciatic nerve pain or numbness, greater trochanteric fracture, and foreign body sensation. The incision healed well without haematoma. There were no significant differences in patient satisfaction (92% and 87%; *P* = 0.532). Specific data are presented in Table 3.

**Table 3:** MRI results at 6 months after surgery, functional Results at last follow-up

Variables	Mesh reconstruction group (*n* = 51 Hips)	Conventional capsular repair group (*n* = 55 Hips)		*P* value
	*n* (%) or Mean ± standard deviation			
Follow up time (month)*	19 ± 3.1	18 ± 3.3	t = 1.409	0.162
Capsular repair in MRI^†^			*χ*^2^ = 17.026	< 0.001
Successful/failed	50 (98%)/1 (2%)	37 (67%)/18 (33%)		
Dislocation†			*χ*^2^ = 2.863	0.244
Yes/no	0 (0%)/51 (100%)	3 (5%)/52 (95%)		
Other complications^†^	0 (0%)	0 (0%)	–	–
Pre-operation HHS*	30 ± 19.8	35 ± 19.4	*t* = − 1.401	0.164
Post-operation HHS*	82 ± 4.8	83 ± 3.9	*t* = − 0.984	0.328
Excellent (≥ 90)	2 (4%)	1 (2%)	*χ*^2^ = 2.468	0.291
Fair (80–89.9)	34 (67%)	44 (80%)		
Good (70–79.9)	15 (29%)	10 (18%)		
Satisfaction^†^			*χ*^2^ = 1.261	0.532
Very satisfied or satisfied	47 (92%)	48 (87%)		
Somewhat satisfied	4 (8%)	6 (11%)		
Somewhat dissatisfied or dissatisfied	0 (0%)	1 (2%)		

HHS, Harris hip score

*The values are given as the mean and standard deviation

^†^The values are given as the number with the percentage in parentheses

Three patients developed hip dislocations, and specific data are presented in Table 4. One dislocation occurred in 87 successfully repaired hips, and two other dislocations occurred in 19 failed repaired hips. There was an association between dislocation and the failure of capsular repair (*P* = 0.026) (Table 5). The relative risk for hip dislocation when capsular repair failed was 9.158-fold greater than when the repair was successful (95% CI, 0.87–95.8).

**Table 4:** The postoperative hip dislocations in three cases

Patients	Age	Sex	Side	Reason for THA	Time	Precipitating factor	Position	Treatment	Capsular repair
1	67	Female	Right	Femoral fracture	Postoperative 7 months	Slip in the shower	Flexion, adduction and internal rotation	Conservative treatment	Successful
2	75	Male	Right	Osteonecrosis	Postoperative 8 months	Sleep after drinking	Flexion, adduction and internal rotation	Conservative treatment	Failed
3	71	Female	Left	Femoral fracture	Postoperative 11 months	Prolonged squat	Excessive flexion	Conservative treatment	Failed

**Table 5:** The correlation between a suture failure and postoperative hip dislocation

	Dislocation	No dislocation	*χ*^2^	*P* value	Relative risk
Failed repair	2	17	4.986	0.026	9.158 (0.87–95.8)
Successful repair	1	86			

## Discussion

We found that mesh reconstruction was associated with a higher rate of posterior capsule integrity and a lower rate of hip dislocation than capsular repair after primary arthroplasty. Our findings suggest that mesh reconstruction provides significant functional benefits and good survival in patients with a high risk of dislocation. What is more gratifying is that mesh reconstruction has not increased the incidence of complications. Compared to capsular repair, mesh reconstruction is relatively complex and takes an additional 12 min to complete. It increases the cost of the treatment but is acceptable.

Previous studies have shown that retention and reconstruction of the hip capsule can significantly reduce the rate of dislocation after primary THA. Pellicci et al. [30] suggested repairing the posterior structures of the hip, which provided a mechanistic block to reduce early dislocation. White et al. [14] performed 1078 hip arthroplasties, and complete posterior capsulectomy was performed in all patients. They found early posterior dislocation in 52 (5%) hips. In another study, they performed posterior capsular repair and hip dislocation occurred in only 3 of 437 (1%) hips. Zhang et al. [31] conducted a meta-analysis (7 clinical trials and 4,594 hips) and found a low dislocation rate after posterior capsular repair. Although capsular repair reduced the dislocation rate, it did not completely eliminate dislocation. Therefore, some surgeons have considered whether the joint capsule remains intact after repair. Allegra et al. [15] found intact capsular structure on MRI one year after surgery in 15 of 32 (48%) patients undergoing posterior capsule repair. Moon et al. [6] reported an 18% failure rate of capsular repair due to insufficient structure and repair under tension and observed a significant correlation between suture failure and dislocation. To better reconstruct the joint capsule, Masterson [18] reconstructed the hip capsule using mesh in 13 patients undergoing tumour resection and hip arthroplasty. Postoperative dislocation occurred in 5 patients. Maslennikov et al. [32] confirmed through finite element analysis that, the strength and stiffness of the closure of the hip capsule defect with mesh were superior to those of interrupted stitches. Mesh has been utilized in surgery because it has excellent tensile strength and decreased foreign-body response compared with other synthetic biomaterials. When the soft tissue is destroyed in orthopaedic surgery, the increased scar tissue formation may enhance the integration of the mesh with the host soft tissue to provide a more lasting reconstruction [17, 32].

In the method we present, mesh was used to reconstruct the capsule while playing a fundamental role in balancing functional mobility and joint stability, such as ischiofemoral ligament [33], which reinforces the capsule during internal rotation in neutral positions as well as in combined flexion-adduction positions [34]. Mesh reconstruction with 90° of hip flexion avoids excessive tension on the reconstructed ligaments. The mesh may irritate the sciatic nerve, causing pain and palsy in the hip. To avoid this complication, we transferred the external rotators over the mesh. We did not need to lengthen the offset to obtain hip stability, which also reduced the risk of sciatic nerve injury [35, 36]. Great trochanteric fracture did not occur in the mesh group. Moreover, the mesh also avoided using restrictive prostheses to reduce the dislocation risk and did not affect prosthesis survival.

Mesh reconstruction is indicated in patients with a high risk of hip dislocation, including neuromuscular disease, degenerative disc disease, lumbosacral fusion, obesity, higher preoperative activity, and poor muscle strength [20–24]. Contraindications are surgical site infection, allergies, cicatrix hyperplasia, and severe osteoporosis.

Mesh reconstruction has advantages. First, the mesh is made from a synthetic polymer material with high strength and good tissue compatibility. Therefore, the reconstruction is stronger than capsular repair and achieves early hip stability. Second, the mesh is located at the posterior aspect of the hip, between the pelvis and the proximal end of the femur, forming a barrier to prevent posterior dislocation. Third, the mesh will twist and tighten to act as a “tension band” and an orbicularis band when the hip flexion is large. Fourth, full range of motion of the hip can be preserved. The disadvantages are the additional time and cost needed. Despite increased time and cost, the decrease in dislocation rates is clinically important, and the benefits of the procedure exceed the disadvantages.

This study has limitations. First, the small sample size produces statistical bias, and the patients with femoral neck fractures led to a high postoperative dislocation rate. Second, the follow-up time was short, and hip function improved over time, even though hip dislocation mostly occurred 120 days after surgery [37]. Third, a further study should be performed to better understand the kinematics of mesh reconstruction. Surgeons’ preference, experience, and ability should increase over time and thus affect results. The operations and assessments were performed at different times, which may have influenced ascertaining the effects of the techniques.

## Conclusion

Compared to conventional capsular repair, mesh reconstruction can maintain the integrity of the hip capsule and decrease the risk of hip dislocation after hip arthroplasty without increasing complications.

## Data Availability

The datasets used and/or analyzed during the current study are available from the corresponding author on reasonable request.
